# Zinc protoporphyrin levels in COVID-19 are indicative of iron deficiency and potential predictor of disease severity

**DOI:** 10.1371/journal.pone.0262487

**Published:** 2022-02-03

**Authors:** Meltem Kilercik, Yasemin Ucal, Muhittin Serdar, Mustafa Serteser, Aysel Ozpinar, Florian J. Schweigert

**Affiliations:** 1 Department of Medical Biochemistry, School of Medicine, Acıbadem Mehmet Ali Aydınlar University, Istanbul, Turkey; 2 Department of Medical Biochemistry, Acibadem Labmed Clinical Laboratories, Istanbul, Turkey; 3 Department of Physiology and Pathophysiology, Institute of Nutritional Science, University of Potsdam, Potsdam, Germany; Oregon State University, UNITED STATES

## Abstract

**Background:**

Coronavirus disease (COVID-19) has a severe impact on all aspects of patient care. Among the numerous biomarkers of potential validity for diagnostic and clinical management of COVID-19 are biomarkers at the interface of iron metabolism and inflammation.

**Methods:**

The follow-up study included 54 hospitalized patients with laboratory-confirmed COVID-19 with a moderate and severe/critical form of the disease. Iron deficiency specific biomarkers such as iron, ferritin, transferrin receptor, hepcidin, and zinc protoporphyrin (ZnPP) as well as relevant markers of inflammation were evaluated twice: in the first five days when the patient was admitted to the hospital and during five to 15 days; and their validity to diagnose iron deficiency was further assessed. The regression and Receiver Operating Characteristics (ROC) analyses were performed to evaluate the prognosis and determine the probability for predicting the severity of the disease in the first five days of COVID-19.

**Results:**

Based on hemoglobin values, anemia was observed in 21 of 54 patients. Of all iron deficiency anemia-related markers, only ZnPP was significantly elevated (P<0.001) in the anemic group. When patients were grouped according to the severity of disease, slight differences in hemoglobin or other anemia-related parameters could be observed. However, the levels of ZnPP were significantly increased in the severely ill group of patients. The ratio of ZnPP to lymphocyte count (ZnPP/L) had a discrimination power stronger than the neutrophil to lymphocyte count ratio (N/L) to determine disease severity. Additionally, only two markers were independently associated with the severity of COVID-19 in logistic regression analysis; D-dimer (OR (5.606)(95% CI 1.019–30.867)) and ZnPP/L ratio (OR (74.313) (95% CI 1.081–5108.103)).

**Conclusions:**

For the first time ZnPP in COVID-19 patients were reported in this study. Among all iron-related markers tested, ZnPP was the only one that was associated with anemia as based on hemoglobin. The increase in ZnPP might indicate that the underlying cause of anemia in COVID-19 patients is not only due to the inflammation but also of nutritional origin. Additionally, the ZnPP/L ratio might be a valid prognostic marker for the severity of COVID-19.

## 1. Introduction

Iron deficiency anemia (IDA) and the anemia of chronic disease (ACD) are the two most common causes of anemia worldwide [1–3]. Anemia can be observed in up to 95% of critically ill patients even if their admission levels were in the normal range. Anemia is of clinical importance in critically ill patients because it is associated with morbidity and mortality. As a consequence of anemia, up to 40% of patients are receiving a red blood cell transfusion during their intensive care unit stay. In COVID-19 patients an association between iron status and the risk of adverse outcomes has been reported showing that patients with low serum iron status and other indicators of iron deficiency anemia are likely to suffer from severe conditions in COVID-19 [4–6]. Therefore it is of concern to manage the anemia in critically ill patients to improve morbidity and mortality [3, 7, 8].

The cause of anemia in critically ill patients is due to many reasons such as nutritional deficiencies, blood loss, or inflammation. Iron deficiency anemia as defined by a decrease in the body´s iron content is one of the main nutritive causes of anemia and usually develops when the absorption of dietary iron in the intestine cannot compensate for an increased iron demand or blood loss [9]. The anemia of chronic disease is also known as functional iron deficiency anemia. ACD has more complex pathophysiology and is due to a variety of clinical conditions such as infections and inflammatory disorders and is driven by inflammatory cytokines. It is commonly defined as a redistribution of iron from the key sites of its utilization to storage sites resulting in decreased iron availability rather than an absolute iron deficiency [1,10]. This results in a sudden onset of anemia in critically ill patients.

Frequently IDA and ACD coexist, rendering the interpretation of erythrocyte indices and parameters of iron status challenging. The differentiation between both disease processes, however, is important to guide diagnosis and treatment. Diagnosis of IDA is based on a variety of laboratory biomarkers related to iron metabolism such as hemoglobin, ferritin, soluble transferrin receptor, zinc protoporphyrin (ZnPP), and hepcidin. The dysregulation of iron homeostasis is differently affecting these biomarkers. Most important is the interplay between iron deficiency and inflammation. Many of the biomarkers are not only affected by iron deficiency but also by inflammation; such as ferritin, a well-known acute-phase protein[11–13]. However, it has been shown that ZnPP is less sensitive to the inflammatory status [14]. In COVID-19 patients some of the iron markers are affected [6, 15]. The causes and consequences are not yet clear to which extend the underlying mechanism is due to the inflammation general or dietary-induced change in iron metabolism.

There is increasing evidence that anemia is also present in COVID-19 patients due to the generally accepted pathophysiological mechanisms occurring in critically ill patients [6, 15, 16]. Based on recent publications, however, there are indications of possible specific interlinks between blood pathologies and COVID-19 suggesting a strong involvement of erythrocytes in the pathophysiology of COVID-19. We addressed the question to which extend the classical biomarkers such as ferritin, soluble transferrin receptor, total iron-binding capacity, transferrin saturation, hepcidin, and serum iron can be used for the diagnosis of iron deficiency anemia, but also ZnPP which is a less frequently used biomarker that, however, is less affected by the severity of inflammation compared to the others [14].

## 2. Methods

### 2.1. Study population

In this study, 54 hospitalized patients with confirmed COVID-19 were admitted to the Acibadem Altunizade Hospital in Istanbul, Turkey from June 1^st^ to August 31^st^, 2020.

A COVID-19 positive case was defined as a positive result to real-time reverse transcriptase polymerase chain reaction (RT-PCR) assay with nasopharyngeal swab specimen. The inclusion criteria were the admission to the hospital with symptoms suggestive of COVID-19 including fever, muscle/joint pain, cough, and sore throat as well as confirmed SARS-COV-2 infection as determined by RT-PCR. All clinical data including exposure history, course of the disease, clinical symptoms, chest CT images, and treatment data were obtained from medical records. Male and non-pregnant female adults of 18 years or older age were included in the study. Patients with malignancy, known previous history of SARS-COV-2 infection, pregnancy as well as those breastfeeding, anticipating discharge from the hospital or transfer to another hospital that was not a study site, were excluded from the study.

The study has been approved by the Ministry of Health of the Republic of Turkey and the Ethics Committee of Acibadem University (ATADEK-2021-04/34). All subjects provided written informed consent.

### 2.2. Sample collection

All the blood samples were collected during two different time points from each patient (<5 days and 5–15 days after hospital admission). Blood samples were collected into blood tubes containing K_2_EDTA (BD Plus-Plastic tubes). All the samples were kept at -80°C until analysis.

### 2.3. Complete blood count (CBC) and other hematological analyses

The complete blood count was performed on Sysmex XN 3000 automated hematology analyzer (Sysmex Co., Kobe, Japan) as employed in routine practice. The analytes and their measurement methods are given in S1 Table.

### 2.4. Zinc protoporphyrin (ZnPP) measurement using HPLC

Zinc protoporphyrin, protoporphyrin, and mesoporphyrin are light-sensitive, therefore exposure to light was kept to a minimum. The sample preparation procedure was adapted from Hart et al. [17]. Briefly, test tubes including 100 μl whole blood samples (EDTA) or external standards were used. Ten μl internal standard (1 mg mesoporphyrin to 10 ml DMSO) and 300 μl extraction buffer (4:1 ethyl acetate/acidic acid(v/v)) were added into the test tubes. Tubes were centrifuged for 10 min at 10000xg and supernatant was collected. Twenty μl of collected supernatant were injected into the HPLC system (Thermo Scientific Dionex UltiMate 3000 FLD-3000, MA USA). Chromatographic studies were performed using a Hypersil GOLD reversed-phase chromatographic column (250 x 4.6 mm, 5 μm particle size) from Thermo Fischer Scientific, USA, and a guard column. A mobile phase of methanol and ammonium acetate (76:24 v/v, pH 7.2) was pumped at a flow rate of 1.4 ml/min at 33°C. The fluorescence detection was with an excitation wavelength of 420 nm and emission at 588 nm.

### 2.5. Patient classification related to anemia

According to WHO, anemia is defined as a hemoglobin level of less than 7.45 mmol/l in non-pregnant women and less than 8.07 mmol/l in men [18]. Patients were grouped as “anemia” and “control” based on the hemoglobin levels. Patients in the anemia group had Hb levels of less than 8.07 mmol/L, and the control group had Hb levels higher than 8.07 mmol/L.

### 2.6. Patient classification related to COVID-19 severity

Patients were further grouped into two groups: Severity Group 1 (Moderate-Inpatient) and Severity Group 2 (Critical-Intensive care) under the criteria set out in the COVID-19 guidelines of the Republic of Turkey, Ministry of Health. Briefly, patients with symptoms such as fever, muscle/joint pain, cough, and sore throat as well as the respiratory rate of <30/minute and SpO_2_ >90 on room air with mild-to-moderate pneumonia findings on lung X-Ray and/or CT scan were grouped as Severity Group 1. Patients with symptoms including fever, muscle/joint pain, cough, and sore throat along with the respiratory rate of >30/minute and SpO_2_ <90 on room air with bilateral diffused pneumonia findings in lung X-ray or CT scan were grouped as Group 2. Based on the classification related to severity; 35 patients were grouped in Severity Group 1 and 19 patients were grouped in Severity Group 2.

### 2.7. Statistical analysis

Continuous variables, presented as the median (25th and 75th percentiles), were compared with the Wilcoxon Mann Whitney test. Receiver operating characteristic (ROC) analyses were performed to test potential anemia markers in COVID-19 to assess severity. Spearman rank correlation was used to evaluate the correlation between variables. A P-value of less than 0.05 was considered statistically significant. The aforementioned statistical analyses were performed using Analyse-it (v4.20.1) software. To test the predictive value of different independent variables for disease severity, we performed multivariate binary logistic regression analysis that was adjusted for age and gender using IBM SPSS 24. In logistic regression analysis, the odd ratios and 95% confidence intervals (CIs) were calculated based on the Box-Cox transformed laboratory parameters.

## 3. Results

### 3.1. Demographic and clinical characteristics of patients with COVID-19

The study included 54 hospitalized subjects diagnosed with COVID-19. The subjects were further categorized according to disease severity; 35 patients (11 female and 24 male) in Severity Group 1 and 19 patients (3 female and 16 male) in Severity Group 2. The mean (±SD) age of subjects in Group 1 (51.97±16.77) and Group 2 (51.84±16.35) showed similar distribution (P>0.05). Hypertension, diabetes mellitus, and ischemic heart disease were presented as co-morbidities among the subjects, with non-significant differences between severity groups (P>0.05). There were no reported deaths in COVID-19 subjects that were included in the study. The demographic and clinical data of the subjects are given in Table 1.

**Table 1:** Demographic and clinical data of the subjects.

Variables	COVID-19 Severity		P value
	Mild to Moderate (Group 1)	Severe/Critical (Group 2)	
**Age (Mean±SD, years)**	51.97±16.77	51.84±16.35	>0.05
**Sex (Number, %)**			>0.05
Female	11 (31.4%)	3 (15.8%)	
Male	24 (68.6%)	16 (84.2)	
**Co-morbidity (Number, %)**			>0.05
None	20 (57.1%)	8 (42.1%)	
HTN	8 (22.9%)	5 (26.3%)	
DM	2 (5.7%)	2 (10.5%)	
COPD	1 (2.9%)	1 (5.3%)	
Other	4 (11.4%)	3 (15.8%)	

**Abbreviations**: COPD, chronic obstructive pulmonary disease; DM, Diabetes Mellitus; HTN, hypertension.

### 3.2. Iron deficiency anemia related laboratory parameters in patients with COVID-19

In a first step, all patients at the two-time points were grouped according to their anemia status (Table 2). The WHO cut-off of 8.07 mmol/L was used for grouping. On both time points, 33 of the patients were classified as non-anemic and 21 as anemic. The median (25^th^-75^th^ percentile) hemoglobin values of control and anemic groups were 8.75 (8.49–9.15) vs. 7.39 (6.35–7.82) and 9.12 (8.25–9.31) vs. 7.26 (6.50–7.54) mmol/L, respectively (P<0.0001). A similar difference was observed for the hematocrit (P<0.0001). No significant differences were observed between control and anemic patients at both time-points for the biomarker of iron deficiency such as ferritin, soluble transferrin receptor, total iron-binding capacity, transferrin saturation, and serum iron. The only significantly different parameter related directly to anemia was ZnPP. At both time points (< 5 days and 5–15 days), median (25^th^-75^th^ percentile) ZnPP levels in anemic patients were significantly higher (P<0.001) compared to non-anemic patients (27.5 (20.63–38.20) vs. 62.5 (43.45–67.46) and 30 (23.15–41.88) vs. 56.85 (37.05–76.96) μmol/mmol heme, respectively). Iron levels between anemic and non-anemic groups were similarly lower (approx. 5 μmol/L) in the group < 5 days compared to the group 5–15 days. In this case in the anemic group, the median (25^th^-75^th^ percentile) levels were significantly lower (P< 0.005) compared to non-anemic (9. 22 (6.71–11.72) and 16.11 (8.77–19.73) μmol/L, respectively).

**Table 2:** Statistical differences between control and anemia subjects in two different time points (< 5 days and 5–15 days) based on CBC and other hematological parameters.

Analyte (units)	< 5 days			5–15 days		
	Control (n = 33)	Anemia (n = 21)	p value	Control (n = 33)	Anemia (n = 21)	p value
	Median (25th-75th Percentile)	Median (25th-75th Percentile)		Median (25th-75th Percentile)	Median (25th-75th Percentile)	
Iron, Serum (μmol/l)	4.48 (2.33–7.97)	3.76 (1.97–7.88)		16.11 (8.77–19.73)	9.22 (6.71–11.72)	**
Ferritin (mg/l)	345 (154.75–657.25)	450 (159.25–1062.75)		332 (239.75–633.25)	372 (134.25–1000.50)	
Hepcidin (mg/l)	47.50 (32.00–67.08)	48.9 (23.83–71.85)		33.5 (24.33–39.20)	30.7 (14.45–58.90)	
Ferritin (mg/l) / Hepcidin (mg/l)	7.26 (4.37–15.06)	9.86 (6.09–18.22)		11.18 (8.04–22.19)	16.54 (6.62–24.48)	
soluble Transferrin receptor(mg/l)	1.23 (1.05–1.41)	1.13 (0.94–1.38)		1.22 (1.11–1.37)	1.19 (0.97–1.42)	
Iron Binding Capacity Total (μmol/l)	43.77 (38.49–49.85)	37.41 (31.06–45.42)		44.57 (41.08–52.00)	37.05 (29.80–50.57)	
Transferrin Saturation (%)	10.2 (5.15–19.05)	7.2 (4.93–32.58)		30.4 (19.48–46.50)	23.85 (17.50–36.35)	
Zinc Protoporphyrin (μmol/molHb)	27.5 (20.63–38.20)	62.5 (43.45–67.46)	****	30 (23.15–41.88)	56.85 (37.05–76.96)	<0.001
C-Reactive Protein (mg/l)	30.7 (11.55–88.85)	78.5 (18.30–110.05)		8.6 (3.08–21.03)	24.45 (8.35–53.60)	*
D-Dimer (μg/ml)	0.55 (0.34–0.79)	1.09 (0.59–2.05)		0.7 (0.46–1.87)	0.99 (0.75–13.53)	
Interleukin-6 (pg/ml)	8.51 (5.78–22.60)	21.8 (10.01–56.63)	*	3.17 (2.00–13.85)	19.05 (6.39–34.10)	*
Plateletcrit (%)	0.22 (0.17–0.25)	0.2 (0.15–0.29)		0.3 (0.24–0.33)	0.33 (0.23–0.38)	
Antibody-synthesizing lymphocytes (%)	0 (0–0.45)	0.45 (0–0.70)		0 (0–0.73)	0 (0–0.40)	
Antibody-synthesizing lymphocytes (cells/L)	0 (0–0.04)	0.03 (0–0.09)		0 (0–0.03)	0 (0–0.04)	
Basophil (%)	0.3 (0.20–0.73)	0.2 (0.10–0.30)	*	0.5 (0.30–0.63)	0.3 (0.20–0.50)	
Eosinophil (%)	0.9 (0.08–1.63)	0.2 (0.10–0.83)		1.6 (0.50–2.00)	0.7 (0.23–1.28)	*
Hematocrit (%)	40.9 (39.35–43.10)	34.5 (32.33–37.18)	****	42 (39.18–44.05)	35.9 (32.90–37.85)	****
High Fluorescence reticulocytes (%)	1.1 (0.30–1.55)	1.5 (0.88–2.48)		1.3 (0.38–2.03)	2.4 (1.88–5.88)	***
Hemoglobin (mmol/l)	8.75 (8.49–9.15)	7.39 (6.35–7.82)	****	9.12 (8.25–9.31)	7.26 (6.50–7.54)	****
Ferritin (mg/l) /Hemoglobin (mmol/l)	24.82 (11.06–44.45)	35.08 (12.77–100.66)	****	25.34 (16.10–41.56)	36.47 (11.37–94.93)	****
Immature reticulocyte fraction (%)	7.75 (4.65–10.20)	9.3 (8.05–12.25)		7.8 (4.25–11.15)	13.6 (11.60–18.85)	**
Low fluorescence reticulocytes (%)	92 (89.80–94.95)	90.7 (87.75–91.95)		92.2 (88.85–95.75)	86.4 (81.15–88.40)	**
Lymphocyte (%)	17.6 (12.70–32.83)	14.6 (9.05–18.55)		27.3 (21.10–36.45)	19.3 (14.15–23.60)	**
Percentage of macrocytic RBCs (%)	3.95 (3.80–4.20)	3.8 (3.30–3.95)	**	4.1 (3.80–4.20)	3.7 (3.45–4.03)	
Mean Corpuscular Hemoglobin (fmol)	1.84 (1.79–1.88)	1.76 (1.69–1.84)	**	1.84 (1.80–1.90)	1.76 (1.67–1.82)	***
Mean Corpuscular Hemoglobin Concentration (g Hb/l)	344 (338.75–351.00)	337 (326.5–340.5)	***	345 (335.50–352.00)	325 (319.75–333.75)	****
Mean Corpuscular Volume (fl)	85.7 (83.38–87.98)	83.7 (80.85–87.43)		86 (84.43–89.50)	85.4 (82.73–88.28)	
Medium fluorescence reticulocytes (%)	6.55 (4.60–8.90)	7.7 (6.78–9.88)	*	6.6 (3.78–9.50)	11.6 (8.80–14.00)	***
Percentage of microcytic RBCs (%)	2.15 (1.50–2.90)	3.45 (2.90–5.55)	**	2.05 (1.35–2.40)	3.1 (2.03–4.20)	**
Monocyte (%)	8.4 (5.43–10.80)	7.2 (5.40–9.55)		8.5 (7.08–10.00)	7.5 (5.55–10.25)	
Mean Platelet Volume (fL)	10.7 (10.00–11.20)	11.2 (9.80–11.95)		10.4 (9.58–11.10)	10.6 (10.08–11.28)	
Neutrophil Lymphocyte Ratio	4.19 (1.58–620)	5.19 (4.14–9.56)		2.23 (1.52–3.15)	3.72 (2.81–5.32)	**
Neutrophil Monocyte Ratio	7.18 (4.83–12.86)	10.19 (7.59–15.57)		6.48 (5.41–8.45)	9.63 (6.18–13.58)	*
Neutrophil (%)	69.8 (54.30–79.78)	76.5 (71.15–84.73)	*	60.4 (53.45–66.48)	72.2 (66.63–76.50)	**
Neutrophil Granularity Intensity (SI)	149 (143.40–153.15)	148.2 (142.40–152.10)		149.7 (145.53–153.80)	148.7 (142.80–151.90)	
Neutrophil Reactivity Intensity (FI)	49.2 (47.30–52.05)	49.65 (46.50–53.90)		45.1 (44.18–47.60)	47.8 (43.90–51.50)	
Platelet-large cell ratio (%)	31.2 (24.73–34.63)	34.4 (23.40–40.48)		28.5 (22.08–34.08)	29.10 (25.68–34.68)	
Platelet Distribution Width (fL)	12.5 (11.30–13.85)	13.2 (10.58–15.60)		12.4 (10.75–13.70)	12.10 (10.88–14.65)	
Platelet (10^9/l)	196 (164.25–249.25)	166 (133.50–279.75)		275 (216.50–321.75)	306 (222.75–391.75)	
Red Blood Cell Count (10^9/l)	4.8 (4.60–5.10)	4.2 (3.83–4.41)	****	4.9 (4.62–5.05)	4.08 (3.79–4.54)	****
Red blood cell hemoglobin content (fmol)	1.86 (1.82–1.91)	1.79 (1.65–1.83)	***	1.86 (1.84–1.91)	1.76 (1.72–1.82)	***
Red blood cell distribution width coefficient of variation (%)	12.6 (12.00–13.20)	13.9 (13.08–15.18)	***	12.8 (12.20–13.20)	13.8 (12.90–15.10)	***
Red blood cell distribution width standard deviation (fl)	39.8 (37.43–41.18)	42.6 (39.55–45.15)	*	39.4 (37.35–42.50)	42.8 (40.00–45.45)	**
Total reactive lymphocytes (%)	0.9 (0.53–1.45)	1.15 (0.65–1.55)		1.00 (0.70–1.58)	1.6 (1.20–2.65)	*
Total reactive lymphocytes (cells/l)	0.08 (0.03–0.11)	0.07 (0.05–0.11)		0.06 (0.04–0.07)	0.13 (0.08–0.16)	*
Percentage of reticulocyte (%)	0.76 (0.56–1.19)	0.82 (0.54–1.06)		0.98 (0.71–1.30)	1.17 (0.80–1.54)	
Absolute reticulocyte count (10^9/l)	0.04 (0.03–0.06)	0.03 (0.02–0.04)		0.05 (0.03–0.07)	0.05 (0.04–0.06)	
Reticulocyte hemoglobin equivalent (fmol)	1.87 (1.77–2.00)	1.76 (1.55–1.94)	**	2.05 (1.96–2.14)	1.94 (1.77–2.04)	**
White Blood Cell Count (10^9/l)	6.09 (4.90–7.95)	5.4 (4.42–7.60)		6.08 (5.07–8.23)	6.07 (5.01–10.22)	

Significance level was set at:

*P<0.05,

**P<0.01,

***P<0.001,

****P< 0.0001.

With regard to all the other parameters, only interleukin-6 showed a significant difference between non-anemic and anemic patients, with significantly higher values in the anemic group (P<0.05). This difference was even more pronounced at the later time-point (P<0.01).

#### 3.2.1 Relationship between different stages of COVID-19 and laboratory parameters

In a second step, all patients at the two-time points were grouped according to their severity of diseases in moderate (Group 1) and severe (Group 2) (Tables 3 and 4) to assess the ability of different parameters to distinguish COVID-19 severity. The grouping was based on the above-described criteria in the Method section. On both time points, 35 of the patients were classified as moderate and 19 as severe.

**Table 3:** Complete blood count and other hematological analysis based on COVID-19 severity (< 5 days).

Variables	COVID-19 Severity						p value
	Mild to Moderate (Group 1; n = 35)			Severe/Critical (Group 2; n = 19)			
	Median	25^th^ Percentile	75^th^ Percentile	Median	25^th^ Percentile	75^th^ Percentile	
White Blood Cell Count (10^9/l)	5.68	4.49	7.02	7.59	4.96	10.91	*
Neutrophil (%)	63.20	55.10	74.85	84.10	78.35	89.95	****
Lymphocyte (%)	26.50	14.80	33.95	10.10	6.35	12.50	
Monocyte (%)	9.00	7.20	10.75	5.20	3.30	7.85	
Eosinophil (%)	0.90	0.15	1.65	0.10	0.05	0.45	
Basophil (%)	0.30	0.20	0.65	0.20	0.05	0.30	
Neutrophil Lymphocyte Ratio	2.34	1.56	5.02	8.55	6.09	14.57	****
Neutrophil Monocyte Ratio	7.18	5.27	9.20	16.39	10.18	26.97	****
Red Blood Cell Count (10^9/l)	4.62	4.37	4.91	4.40	4.07	4.74	
Hemoglobin (mmol/l)	13.70	12.85	14.50	12.70	11.15	14.00	
Hematocrit (%)	39.80	37.60	41.95	37.10	34.05	40.30	
Mean Corpuscular Volume (fl)	85.60	83.20	87.45	83.80	82.60	87.65	
Mean Corpuscular Hemoglobin (fmol)	29.40	28.30	30.20	29.00	28.10	29.65	
Mean Corpuscular Hemoglobin Concentration (g Hb/l)	34.30	33.50	34.90	34.10	32.95	34.55	
Red blood cell distribution width standard deviation (fl)	39.80	37.75	41.95	42.50	40.00	44.45	*
Red blood cell distribution width coefficient of variation (%)	12.80	12.40	13.45	13.40	13.05	14.75	*
Platelet (10^9/l)	205.00	158.00	279.00	166.00	139.50	210.50	
Mean Platelet Volume (fl)	10.70	9.95	11.20	11.30	10.30	11.85	*
Platelet Distribution Width (fl)	12.40	10.60	13.80	14.00	11.40	14.90	
Platelet-large cell ratio (%)	30.00	24.05	34.15	34.70	28.10	39.30	
Plateletcrit (%)	0.23	0.17	0.28	0.19	0.16	0.25	
Percentage of macrocytic RBCs (%)	3.85	3.60	4.10	3.95	3.73	4.13	
Percentage of microcytic RBCs (%)	2.30	1.70	3.20	3.40	2.20	4.20	
Red blood cell hemoglobin content (fmol)	29.80	28.88	30.40	29.25	28.60	30.13	
Percentage of reticulocyte (%)	0.76	0.55	0.99	0.84	0.59	1.51	
Absolute reticulocyte count (10^9/l)	0.04	0.03	0.04	0.03	0.03	0.06	
Immature reticulocyte fraction (%)	7.75	4.48	10.15	9.70	8.25	10.30	*
Low fluorescence reticulocytes (%)	92.00	89.80	94.98	90.30	89.70	91.75	
Medium fluorescence reticulocytes (%)	6.55	4.50	8.78	7.90	7.10	9.40	**
High Fluorescence reticulocytes (%)	1.10	0.30	1.65	1.50	0.80	2.35	*
Reticulocyte hemoglobin equivalent (fmol)	30.10	28.30	32.20	29.20	26.70	32.05	
Antibody-synthesizing lymphocytes (cells/l)	0.00	0.00	0.04	0.01	0.00	0.06	
Antibody-synthesizing lymphocytes (%)	0.00	0.00	0.55	0.15	0.00	0.50	
Total reactive lymphocytes (cells/l)	0.07	0.05	0.11	0.06	0.02	0.11	
Total reactive lymphocytes (%)	1.30	0.85	1.70	0.60	0.40	0.78	
Neutrophil Reactivity Intensity (FI)	49.00	47.23	51.68	50.90	47.08	54.03	
Neutrophil Granularity Intensity (SI)	149.00	145.23	150.98	148.20	141.78	153.55	
Interleukin-6 (pg/ml)	10.30	5.91	16.70	33.50	12.08	60.10	**
Iron, Serum (μmol/l)	26.00	13.75	43.25	15.00	12.00	47.00	
Iron Binding Capacity Total (μmol/l)	234.00	205.50	263.50	237.00	208.50	283.00	
Transferrin Saturation (%)	10.74	5.89	21.27	6.79	4.52	27.61	
Ferritin (mg/l)	345.00	139.00	586.00	621.00	267.00	1305.50	*
Hepcidin (mg/l)	39.10	28.55	63.90	65.20	33.15	77.75	
soluble Transferrin receptor (mg/l)	1.23	1.06	1.38	1.11	0.95	1.43	
D-Dimer (μg/ml)	0.38	0.27	0.68	1.13	0.58	2.03	****
C-Reactive Protein (mg/l)	2.28	1.12	7.98	10.45	2.83	20.99	**
Zinc Protoporphyrin (μmol/molHb)	33.67	21.27	47.91	44.98	31.23	67.70	*

Significance level was set at:

*p<0.05,

**p<0.01,

***p<0.001,

****p< 0.0001.

**Table 4:** Complete blood count and other hematological analysis based on COVID-19 severity (5–15 days).

Variables	COVID-19 Severity						p value
	Mild to Moderate (Group 1; n = 35)			Severe/Critical (Group 2; n = 19)			
	Median	25^th^ Percentile	75^th^ Percentile	Median	25^th^ Percentile	75^th^ Percentile	
White Blood Cell Count (10^9/L)	5.89	4.81	7.38	7.60	5.80	12.82	**
Neutrophil (%)	62.30	54.65	67.10	74.50	65.50	85.65	***
Lymphocyte (%)	26.60	22.70	33.10	17.10	7.95	21.25	
Monocyte (%)	8.50	7.50	10.00	7.20	5.10	9.95	
Eosinophil (%)	1.60	0.90	2.00	0.30	0.00	1.15	
Basophil (%)	0.50	0.40	0.60	0.30	0.10	0.45	
Neutrophil Lymphocyte Ratio	2.24	1.68	2.96	4.17	3.24	11.01	****
Neutrophil Monocyte Ratio	6.84	5.53	8.73	9.78	6.33	16.76	**
Red Blood Cell Count (10^9/l)	4.90	4.34	5.00	4.46	3.82	4.73	
Hemoglobin (mmol/l)	13.70	12.35	14.90	12.10	10.95	13.70	
Hematocrit (%)	40.80	38.00	43.10	37.50	35.00	39.95	
Mean Corpuscular Volume (fl)	85.20	83.25	89.25	86.10	85.20	88.75	
Mean Corpuscular Hemoglobin (fmol)	29.40	28.10	30.35	29.00	28.35	29.85	
Mean Corpuscular Hemoglobin Concentration (g Hb/l)	34.00	32.75	34.90	33.10	32.25	34.70	
Red blood cell distribution width standard deviation (fl)	39.40	37.30	42.55	43.20	39.85	45.55	**
Red blood cell distribution width coefficient of variation (%)	12.80	12.40	13.20	13.50	13.05	15.35	**
Platelet (10^9/l)	287.00	223.00	374.00	282.00	189.50	321.00	
Mean Platelet Volume (fl)	10.10	9.55	10.85	10.90	10.35	11.55	**
Platelet Distribution Width (fl)	11.50	10.60	13.35	13.40	11.55	14.95	**
Platelet-large cell ratio (%)	25.70	2205	32.00	32.60	27.65	37.45	**
Plateletcrit (%)	0.30	0.25	0.36	0.31	0.22	0.34	
Percentage of macrocytic RBCs (%)	4.00	3.60	4.10	4.05	3.65	4.33	
Percentage of microcytic RBCs (%)	2.20	1.70	3.10	2.25	1.80	3.23	
Red blood cell hemoglobin content (fmol)	29.70	28.45	30.45	29.35	28.50	30.18	
Percentage of reticulocyte (%)	1.05	0.84	1.31	1.02	0.71	1.69	
Absolute reticulocyte count (10^9/l)	0.05	0.04	0.06	0.05	0.03	0.08	
Immature reticulocyte fraction (%)	9.70	5.05	13.85	12.40	8.80	14.60	
Low fluorescence reticulocytes (%)	90.30	86.15	94.95	87.60	85.40	91.20	
Medium fluorescence reticulocytes (%)	7.90	3.95	11.60	10.40	7.25	12.10	
High Fluorescence reticulocytes (%)	1.50	0.45	2.45	2.00	1.10	2.95	
Reticulocyte hemoglobin equivalent (fmol)	32.20	30.75	33.75	32.90	31.30	35.05	
Antibody-synthesizing lymphocytes (cells/l)	0.00	0.00	0.02	0.03	0.00	0.10	*
Antibody-synthesizing lymphocytes (%)	0.00	0.00	0.28	0.25	0.01	0.93	*
Total reactive lymphocytes (cells/l)	0.06	0.05	0.09	0.13	0.07	0.20	*
Total reactive lymphocytes (%)	1.25	0.80	1.98	1.30	0.95	2.60	
Neutrophil Reactivity Intensity (FI)	45.60	44.00	47.60	47.70	44.48	52.33	
Neutrophil Granularity Intensity (SI)	149.40	145.60	153.50	149.30	144.33	152.78	
Interleukin-6 (pg/ml)	3.10	2.00	11.35	28.90	14.50	54.05	****
Iron, Serum (μmol/l)	60.50	49.00	94.75	58.00	37.00	96.50	
Iron Binding Capacity Total (μmol/l)	262.00	233.25	301.50	200.00	177.00	244.50	
Transferrin Saturation (%)	26.89	18,04	34.14	35.48	19.56	48.51	
Ferritin (mg/l)	321.00	166,00	476.50	915.00	231.50	1612.50	*
Hepcidin (mg/l)	28.50	19,38	37.90	43.30	30.50	56.85	**
soluble Transferrin receptor(mg/l)	1.24	1,11	1.41	1.20	0.95	1.31	
D-Dimer (μg/ml)	0.54	0,22	0.74	0.99	0.79	2.45	**
C-Reactive Protein (mg/l)	0.69	0,25	1.60	2.93	1.01	9.19	**
Zinc Protoporphyrin (μmol/molHb)	36.41	26,11	45.99	55.10	33.25	73.50	*

Significance level was set at:

*P<0.05,

**P<0.01,

***P<0.001,

****P< 0.0001.

**Fig 1:**
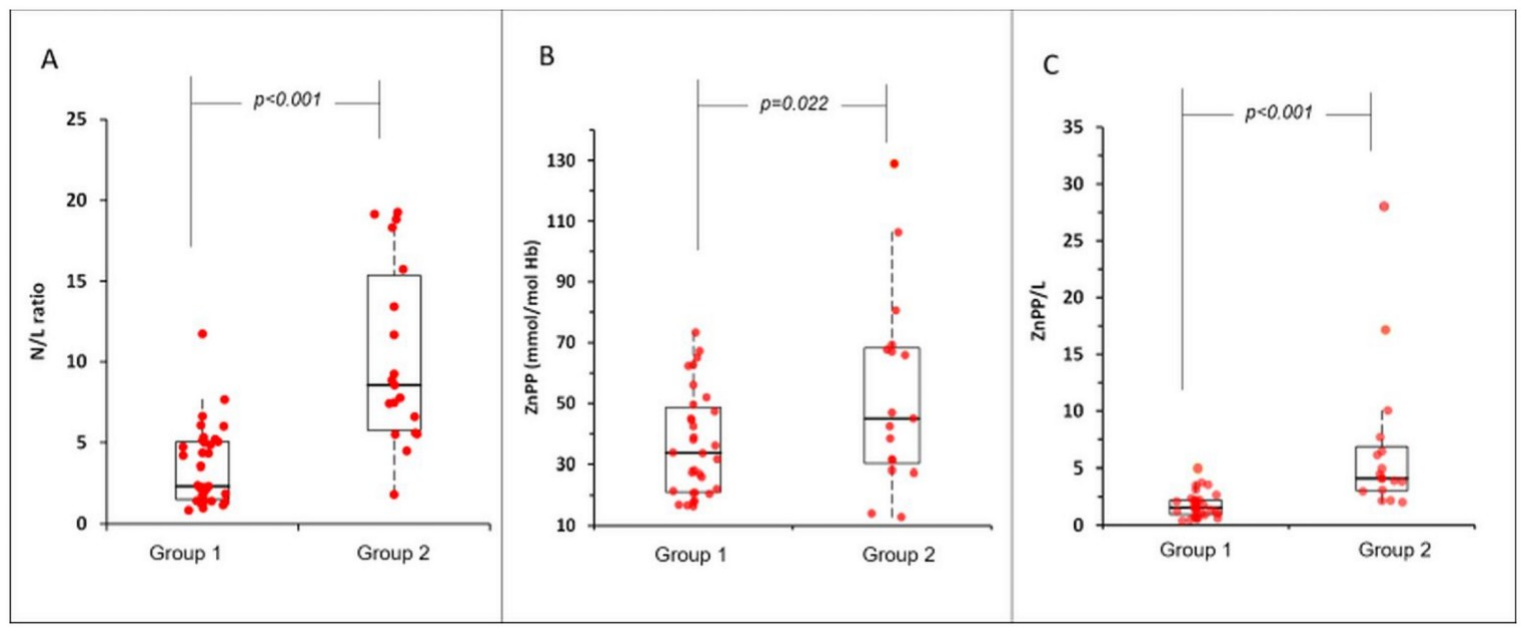
Box plot diagram showing the A. N/L ratio, B. ZnPP (mmol/mol Hb), C. ZnPP/L ratio on COVID-19 severity groups. Group 1: Moderate, Group 2: Severe.

Regarding the parameters related to iron metabolism and inflammation, differences between patients with moderate signs and those with severe signs of the disease were observed with slightly lower median (25^th^-75^th^ percentile) values in the severely affected patients for iron (60.50 (49.00–94.75) and 58.00 (37.00–96.50) μmol/L, respectively) and higher levels for ferritin (321.00 (166.00–476.50) and 915.00 (231.50–1612.50) mg/L, respectively), hepcidin (28.50 (19.38–37.90) and 43.30 (30.50–56.85) mg/L, respectively), D-dimer (0.54 (0.22–0.74) and 0.99 (0.79–2.45) μg/ml, respectively) and CRP (0.69 (0.25–1.60) and 2.93 (1.01–9.19) mg/L, respectively). No differences were observed for hemoglobin between Group 1 and Group 2 (13.70 (12.35–14.90) and 12.10 (10.95–13.70), respectively). Median (25^th^-75^th^ percentile) ZnPP levels in Group 2 (44.98 (30.20–68.24)) were significantly higher than subjects in Group 1 (33.67 (21.08–48.64)) (P<0.05) indicating a notable distinction between COVID-19 severity (Fig 1B).

Similarly, a higher N/L ratio was observed in patients in the severe group (Group 2) compared to patients with moderate disease (Group 1) (Fig 1A).

We then examined the ability of the ZnPP/L ratio to see if the discrimination between COVID-19 severities improves or not in the first five days. This potential marker for disease severity distinction was notable as it included an anemia marker (ZnPP) as well. Median (25^th^-75^th^ percentile) ZnPP/L ratio in Group 2 (4.14 (3.05–6.84)) was significantly higher than subjects in Group 1 (1.55 (0.94–2.22)) (P<0.0001), indicating a remarkable distinction between disease severity groups (Fig 1C). Results show that that the ZnPP/L ratio is better in discriminating severeness than the N/L ratio.

**Fig 2:**
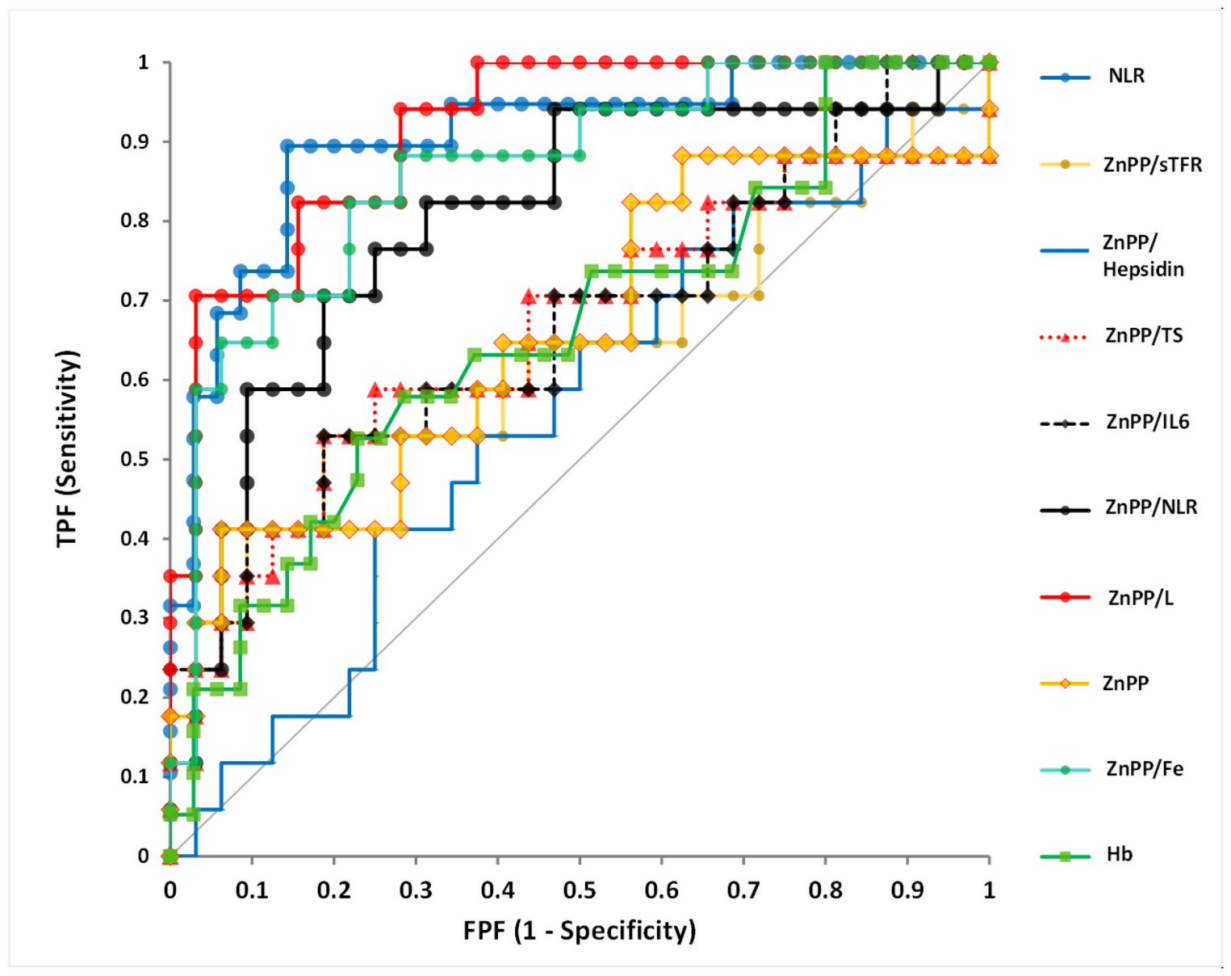
Receiver operating characteristic (ROC) curves displaying the sensitivity and specificity of NLR, ZnPP/sTFR, ZnPP/Hepcidin, ZnPP/TS, ZnPP/IL-6, ZnPP/NLR, ZnPP/L, ZnPP, ZnPP/Fe, Hb on distinguishing COVID-19 severity. NLR: N/L Ratio, ZnPP: Zinc Protoporphyrin, sTFR: soluble Transferrin receptor, TS: Transferrin Saturation; L: Lymphocyte, Hb: hemoglobin.

To further investigate the potential of the ZnPP/L ratio in discriminating COVID-19 severity in the first five days in comparison to the other ratios, we used ROC curve analysis. We built ROC curves for N/L, ZnPP/L, ZnPP, ZnPP/sTFR, ZnPP/hepcidin, ZnPP/TS, ZnPP/IL-6, ZnPP/L, ZnPP/Fe, and Hb. Zinc protoporphyrin and its ratio to specific markers along with CRP and Hb were specifically used to compare the potential of ZnPP in discriminating severity of COVID-19. The highest AUC value was detected for ZnPP/L (AUC:0.92) followed by N/L (AUC:0.90) (Fig 2, Table 5). Additionally, the AUC values for the rest of the biomarkers that showed statistical significance between groups for distinguishing COVID-19 severity were given in S2 Table.

**Table 5:** Receiver operating characteristic (ROC) curve analysis for ZnPP and ZnPP-to-different markers along with NLR and Hb.

	AUC	CI 95%
NLR	0.905	0.82–0.99
ZnPP/sTFR	0.629	0.44–0.81
ZnPP/Hepcidin	0.559	0.39–0.73
ZnPP/TS	0.658	0.48–0.84
ZnPP/IL-6	0.669	0.50–0.84
ZnPP/NLR	0.803	0.67–0.94
ZnPP/L	0.915	0.84–0.99
ZnPP	0.656	0.48–0.83
ZnPP/Fe	0.864	0.75–0.97
Hb	0.661	0.50–0.82

**Abbreviations**: AUC, area under curve; CI, confidence interval; Hb, hemoglobin; L, Lymphocyte; NLR, N/L ratio; sTFR, soluble Transferrin receptor; TS, Transferrin Saturation; ZnPP, Zinc Protoporphyrin.

Finally, we performed logistic regression analysis to evaluate whether different variables were independently associated with COVID-19 severity. Among the infection markers and anemia-related markers, only two markers were independently associated with the severity of COVID-19; namely D-dimer (OR(5.606)(95% CI 1.019–30.867)) and ZnPP/L ratio (OR (74.313) (95% CI 1.081–5108.103)) (Table 6).

**Table 6:** Logistic regression analysis for single predictors associated with COVID-19 severity.

Variables	β	OR	95% CI of OR	
			Lower	Upper
Gender	0.769	2.158	0.043	109.474
Age	1.278	3.590	0.166	77.772
D-dimer	1.724	5.606*	1.019	30.867
NLR	2.462	11.731	0.878	156.747
ZnPP/L	4.308	74.313*	1.081	5108.103
TS	-0.597	0.550	0.026	11.563
Ferritin	0.149	1.160	0.752	1.791
Hepsidin	0.027	1.028	0.865	1.221
sTFR	-0.077	0.926	0.005	187.524
CRP	-0.764	0.466	0.104	2.099
IL-6	0.937	2.554	0.314	20.792
Constant	-12.497			

**Abbreviations**: CRP, C-reactive protein; NLR, N/L ratio; sTFR, soluble Transferrin receptor; TS, Transferrin Saturation; ZnPP, Zinc Protoporphyrin. Significance level was set at:

*p<0.05.

## 4. Discussion

In this study, for the first time, we report on ZnPP in COVID-19 patients. We show that elevated ZnPP levels are indicative of an iron deficiency in anemic patients with COVID-19 diagnosis. We further show that there is a close relationship between increased ZnPP levels and the severity of disease and that the ZnPP/lymphocyte (ZnPP/L) ratio might have the potential to predict the severity of COVID-19, specifically assessed in the first five days when the patients are admitted to the hospital.

Anemia in critically ill patients such as those suffering from COVID-19 is frequently observed. For example, Bellman-Weiler et al. showed that 24.7% of patients were anemic with a high ratio of anemia of inflammation. In the same cited study, the authors indicated that anemia was significantly associated with in-hospital mortality [19]. In another study, the prevalence of anemia was shown to be high (49%) in hospitalized COVID-19 subjects [20]. The cause of anemia is often related to the limited availability of iron (IDA) or a disturbance in iron metabolism because of the inflammation process (ACD) [21]. The diagnostic approach to distinguish ACD from anemia due to iron deficiency is difficult. In IDA, ferritin, transferrin saturation, and ZnPP are generally valid markers of body iron stores. A reduction of ferritin below 30 ng/ml and ZnPP levels above 40 μmol/mol heme are indicative of an absolute IDA [22, 23]. Due to the inflammatory status, ACD is associated with decreased hemoglobin and iron levels, decreased transferrin saturation but an increase in ferritin levels as well as an increase in CRP, IL-6, and hepcidin levels [24].

Many of the currently described changes in COVID-19 patients support the development of an ACD. Already early in the COVID-19 pandemic changes in hemoglobin levels have been reported [5, 25, 26]. A retrospective study concludes that lower hemoglobin levels at admission are associated with a poorer prognosis [27]. Anemia and alteration of iron hemostasis are reported to be highly prevalent in hospitalized COVID-19 patients [19]. However, hemoglobin levels were not affected by the severeness of the disease as has been reported [15, 28]. The reason that we did not observe any changes in hemoglobin levels in Group 1 and Group 2 might be due to the effect of similar ages in our groups. In a recent systematic review and meta-analysis, Taneri et al. showed that hemoglobin levels were lower in older subjects diagnosed with COVID-19 [15]. In our study, 39% of patients were anemic. In another retrospective study, higher values of 68% were observed [19]. Based on low hemoglobin levels and high ferritin levels it has been estimated that more than 56% of anemia cases in COVID-19 patients are due to ACD [26]. In this context, the observed improvement in respiratory symptoms under EPO can be considered as an effect on iron metabolism[29]. On the other hand, it has been suggested to use iron deprivation as a promising adjuvant therapeutic against viral survival. This is based on the need of the virus of iron-containing enzymes for the completion of the replication process[30]. With regard to the disturbance of hemoglobin metabolism, acute respiratory distress syndrome (ARDS) is one of the critical stages of COVID-19 resulting in lung injury and hemolysis. This results in low-level oxygenation, elevation of free iron and the down regulation of heme oxygenase-1 (HO-1) [31]. Based on this observation it is speculated that Hb, HO-1 and iron overload are possible targets for the treatment of COVID-19 [32].

In general, lower hemoglobin levels increase the demand for better-controlled blood management to achieve an optimal patient outcome [33]. A recent paper reports extremely high levels of ferritin in COVID-19 patients in general and especially under hemodialysis [34]. These elevated ferritin levels are due to the fact that ferritin is both, a marker of inflammation and iron status. Thus, the increase is primarily due to the inflammation associated with the infection as supported by a similar increase in the acute phase proteins CRP and IL-6 in this study and related ones [35, 36]. However, the ferritin levels in these patients were not correlated with the classical acute-phase protein CRP. This could indicate that in this case, ferritin is indicating a change in iron metabolism [34]. It has, however, been speculated that ferritin might also function as a signal molecule as a direct mediator of the immune system [36].

Recently, hepcidin has been introduced as a diagnostic biomarker for differentiation between IDA and ACD being in the normal range in IDA/ACD and elevated in IDA alone, indicating iron restriction as to the suppressive signal [37]. In COVID-19 patients increased levels of hepcidin were predicting COVID-19 severeness [38]. Despite the fact that hepcidin levels were significantly higher in severely affected patients, in our study even in combination with lymphocyte count ratio hepcidin was of low predictive value. Those differences between our and previous studies concerning ferritin or hepcidin [39] are likely explained by patient heterogeneity such as patients’ comorbidities and baseline demographics.

Different studies have identified ZnPP as a biomarker of IDA which is not or little affected by co-occurring acute inflammations [14, 40]. In ACD ZnPP has been shown to detect and quantify derangements of iron metabolism associated with chronic inflammatory disorders and also helps to monitor the success of iron therapy of chronic inflammatory diseases[23, 41]. The diagnostic value of ZnPP as an indicator of IDA in hospitalized patients has shown that ZnPP has a relatively higher degree of diagnostic efficiency better than iron and ferritin for this patient population. It has thus been proposed that ZnPP may be used as a screening tool for IDA in hospitalized patients [42]. The usability of ZnPP as a biomarker has been validated especially in low-resource settings in which IDA is frequently associated with a different cause of inflammation. As erythropoiesis becomes iron deficient the incorporation of zinc instead of iron into protoporphyrin-IX is mediated by the erythroid enzyme ferrochelatase. Increased ratio of ZnPP/heme indicates IDA and is observed in different forms of anemia such as IDA and ACD [43]. It might be emphasized that ZnPP is a cost-effective and simple to analyse biomarker for ID, especially in hospital settings if the direct measurement in a drop of capillary whole blood is used instead of a complex extraction from blood and later HPLC analysis as it has been performed in our study [44, 45].

This study provides novel data reporting the ZnPP measurements in COVID-19 subjects. However, our study is limited in that ZnPP levels in males and females were not individually reported due to the restrictions in sample size. In conclusion, ZnPP can serve as a biomarker for the diagnosis of causes of anemia in hospital settings but might have the potential to be a predictor of COVID-19 severity when used in combination with lymphocyte count due to the stronger discrimination power than the N/L ratio. Letter one might indicate a direct involvement of iron deficiency in COVID-19 pathogenesis. These conclusions, however, need to be confirmed in further studies in different settings that include subjects with IDA and anemia.

## Supporting information

S1 Table. Measurement methods and analyzers for the analytes measured as part of CBC and other hematological analyses (DOCX)

S2 Table. Receiver operating characteristic (ROC) curve analysis for specific biomarkers (DOCX)

S1 Data Patient data. Minimal dataset are given as a supplementary file and uploaded to the Plosone submission system.
(XLSX)
