# Supplementary material for: Zinc protoporphyrin levels in COVID-19 are indicative of iron deficiency and potential predictor of disease severity
Source: PLoS One. 2022 Feb 3;17(2):e0262487. doi: 10.1371/journal.pone.0262487 (PMC8812978; doi:10.1371/journal.pone.0262487)
Supplement: S1 Table. Measurement methods and analyzers for the analytes measured as part of CBC and other hematological analyses — (DOCX) [file pone.0262487.s001.docx]

| **Analytes (unit)** | **Measurement Methods** | **Analyzers** |
| --- | --- | --- |
| Iron (µmol/l) | Colorimetric | ADVIA Chemistry XPT |
| TIBC (total iron-binding capacity) (µmol/l) | Spectrophotometric | ADVIA Chemistry XPT |
| Transferrin Saturation (%) | Ironx100/TIBC | ADVIA Chemistry XPT |
| Ferritin (mg/l) | Immunoassays | Atellica IM Analyzer |
| Hepcidin (mg/l) | ELISA |  |
| soluble Transferrin receptor (mg/l) | Nephelometry | BN ProSpec |
| C-Reactive Protein (mg/l) | Immunoturbidimetric | ADVIA Chemistry XPT |
| Interleukin-6 (pg/ml) | Immunoassay | IMMULITE 2000 XPi |
| D-Dimer (µg/ml) | Immunoturbidimetric | SYSMEX CS5100 |
| WBC (10^9^ L^-1^) | Fluorescence flow cytometry | Sysmex XN 3000 |
| RBC (10^9^ L^-1^) | Hydro Dynamic focusing (DC detection) | Sysmex XN 3000 |
| Hemoglobin (mmol/l) | SLS-Hemoglobin | Sysmex XN 3000 |
| Hematoctrit (%) |  | Sysmex XN 3000 |
| MCV (fl) | HCT/RBC | Sysmex XN 3000 |
| MCH (fmol) | HGB/RBC | Sysmex XN 3000 |
| MCHC (g) | HGB/HCT | Sysmex XN 3000 |
| Platelet (10^9^ L^-1^) | Hydro Dynamic focusing (DC detection) | Sysmex XN 3000 |
| RDW-SD | obtained from the RBC histogram | Sysmex XN 3000 |
| RDW-CV | obtained from the RBC histogram | Sysmex XN 3000 |
| PDW (fl) | obtained from the PLT histogram | Sysmex XN 3000 |
| MPV (fl) | PCT (%)/PLT count | Sysmex XN 3000 |
| P-LCR (%) | obtained from the PLT histogram | Sysmex XN 3000 |
| PCT (%) |  | Sysmex XN 3000 |
| Neutrophil (%) | Fluorescence flow cytometry | Sysmex XN 3000 |
| Lymphocyte (%) | Fluorescence flow cytometry | Sysmex XN 3000 |
| Monocyte (%) | Fluorescence flow cytometry | Sysmex XN 3000 |
| Eosinophil (%) | Fluorescence flow cytometry | Sysmex XN 3000 |
| Basophil (%) | Fluorescence flow cytometry | Sysmex XN 3000 |
| RE-LYMP (cells/l) | Fluorescence flow cytometry | Sysmex XN 3000 |
| NEUT-GI (SI) | Fluorescence flow cytometry | Sysmex XN 3000 |
| NEUT-RI (FI) | Fluorescence flow cytometry | Sysmex XN 3000 |
| AS-LYMP (cells/l) | Fluorescence flow cytometry | Sysmex XN 3000 |
| RET (%) | Fluorescence flow cytometry | Sysmex XN 3000 |
| IRF (%) | MFR+HFR | Sysmex XN 3000 |
| LFR (%) | Fluorescence flow cytometry | Sysmex XN 3000 |
| MFR (%) | Fluorescence flow cytometry | Sysmex XN 3000 |
| HFR (%) | Fluorescence flow cytometry | Sysmex XN 3000 |
| RBC Hb Content (fmol) | Fluorescence flow cytometry | Sysmex XN 3000 |
| Reticulocyte (fmol) | Fluorescence flow cytometry | Sysmex XN 3000 |
| Macro-R (%) | obtained from the RBC histogram. | Sysmex XN 3000 |
| Micro-R (%) | obtained from the RBC histogram. | Sysmex XN 3000 |
|  |  |  |

**S1 Table:** Measurement methods and analyzers for the analytes measured as part of CBC and other hematological analyses

**Abbreviations:** AS-LYMP, antibody synthesizing lymphocytes; FI, fluorescence intensity; Hb, hemoglobin; HFR, high fluorescence reticulocytes; IRF, immature reticulocyte fraction; LFR, low fluorescence reticulocytes; Macro-R, percentage of macrocytic red cells; MCH, mean corpuscular volume hemoglobin; MCHC, mean corpuscular volume hemoglobin concentration; MCV, mean corpuscular volume; MFR, medium fluorescence reticulocytes; Micro-R, percentage of microcytic red cells; MPV, mean platelet volume; NEUT-GI, neutrophil granularity intensity; NEUT-RI, neutrophil reactivity intensity; PCT, plateletcrit; PDW, platelet distribution width; P-LCR, platelet-large cell ratio; RBC, red blood cell count; RET, percentage of reticulocyte; RE-LYMP, total reactive lymphocytes; SI, scatter intensity; WBC, white blood cell count
