# Supplementary material for: Zinc protoporphyrin levels in COVID-19 are indicative of iron deficiency and potential predictor of disease severity
Source: PLoS One. 2022 Feb 3;17(2):e0262487. doi: 10.1371/journal.pone.0262487 (PMC8812978; doi:10.1371/journal.pone.0262487)
Supplement: S2 Table. Receiver operating characteristic (ROC) curve analysis for specific biomarkers — (DOCX) [file pone.0262487.s002.docx]

**S2 Table:** Receiver operating characteristic (ROC) curve analysis for specific biomarkers

|  | AUC | CI 95% |
| --- | --- | --- |
| IL-6 | 0.738 | 0.59-0.89 |
| TS | 0.553 | 0.37-0.73 |
| Ferritin | 0.669 | 0.51-0.83 |
| Ferritin/Hepcidin | 0.615 | 0.44-0.79 |
| Ferritin /Hb | 0.693 | 0.54-0.85 |
| Hepcidin | 0.617 | 0.45-0.79 |
| sTFR | 0.547 | 0.37-0.73 |
| D-Dimer | 0.819 | 0.70-0.94 |
| CRP | 0.730 | 0.58-0.88 |

**Abbreviations:** AUC, area under curve; CI, confidence interval; CRP, C-reactive protein; Hb, hemoglobin; NLR, N/L ratio; sTFR, soluble Transferrin receptor; TS, Transferrin Saturation
